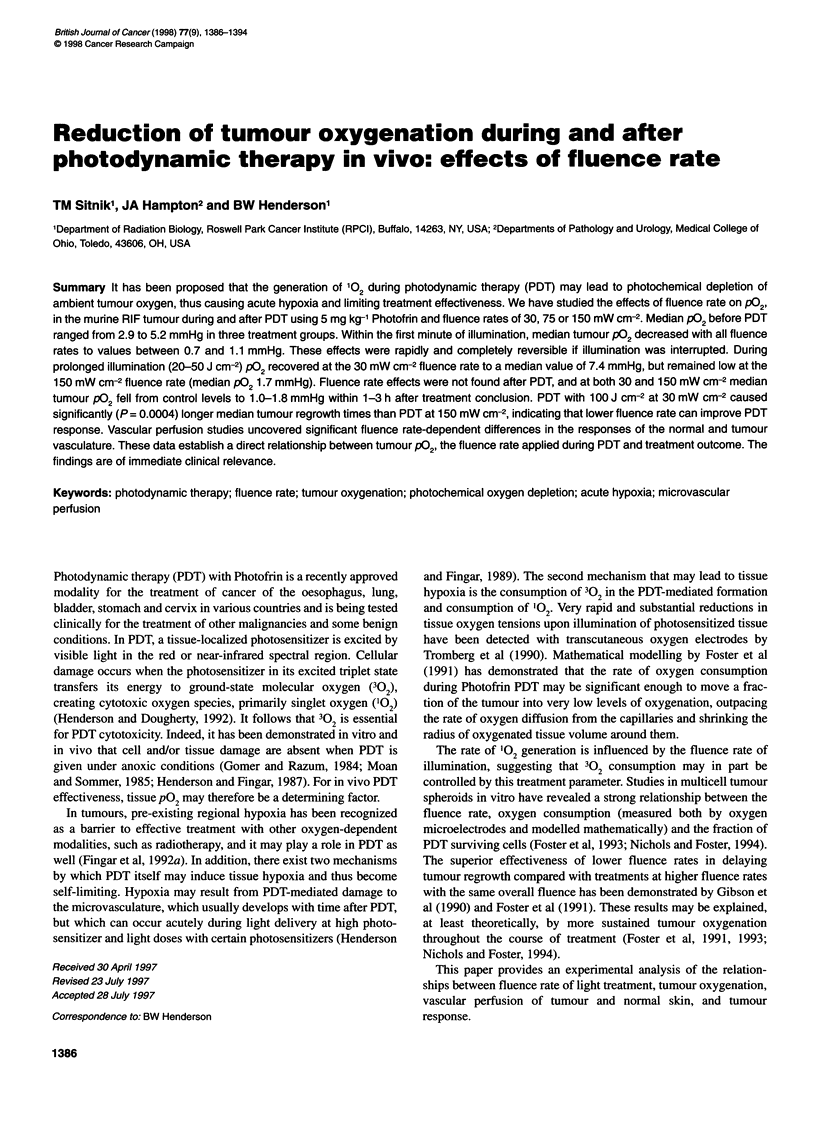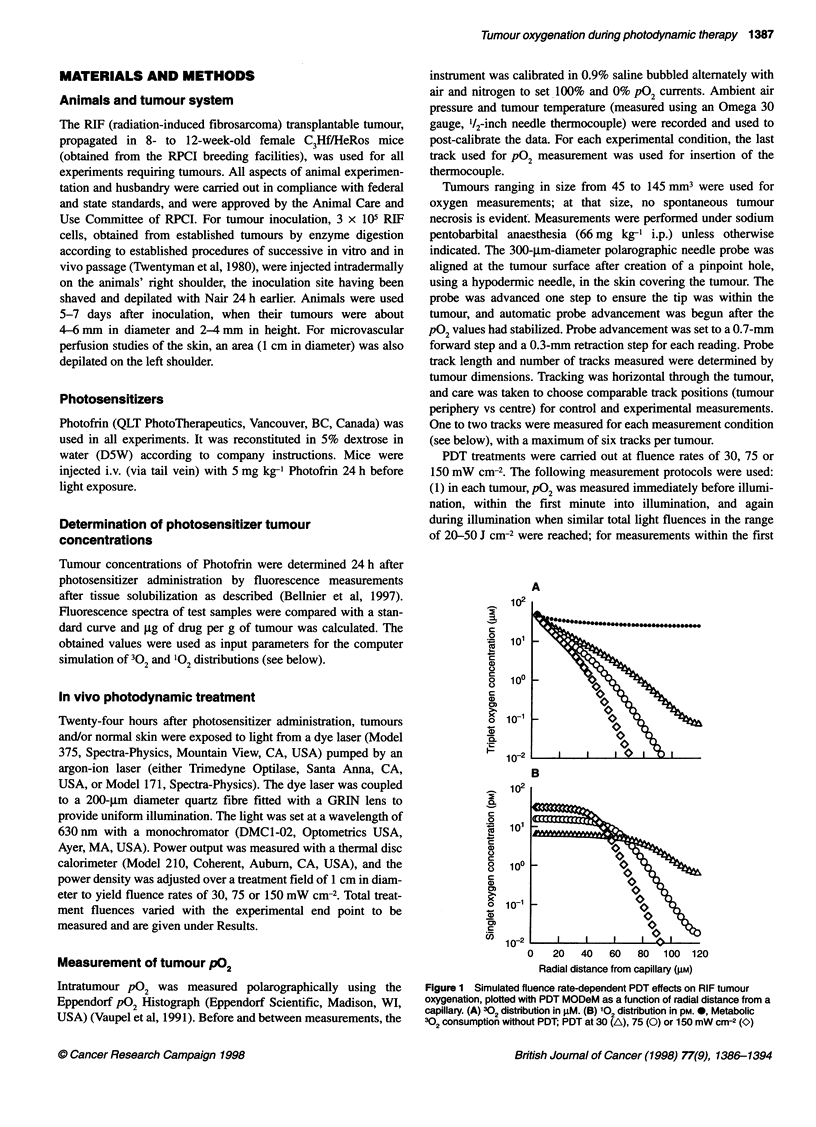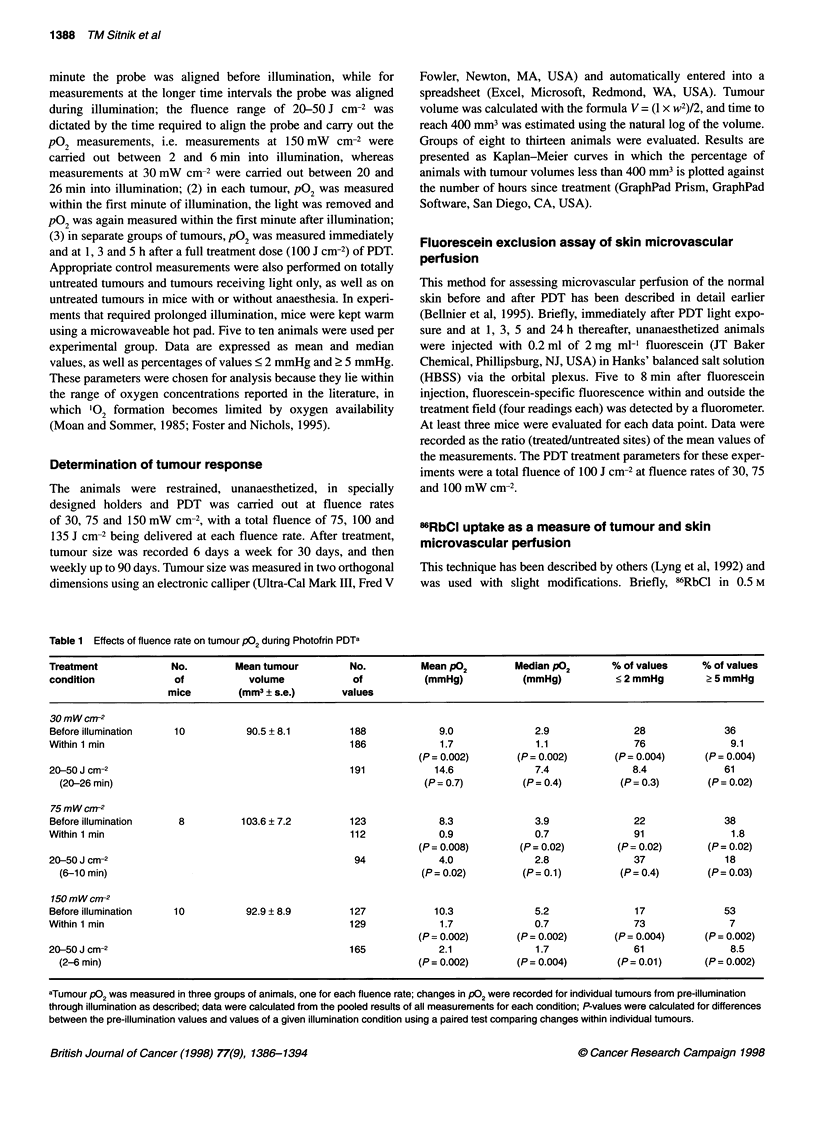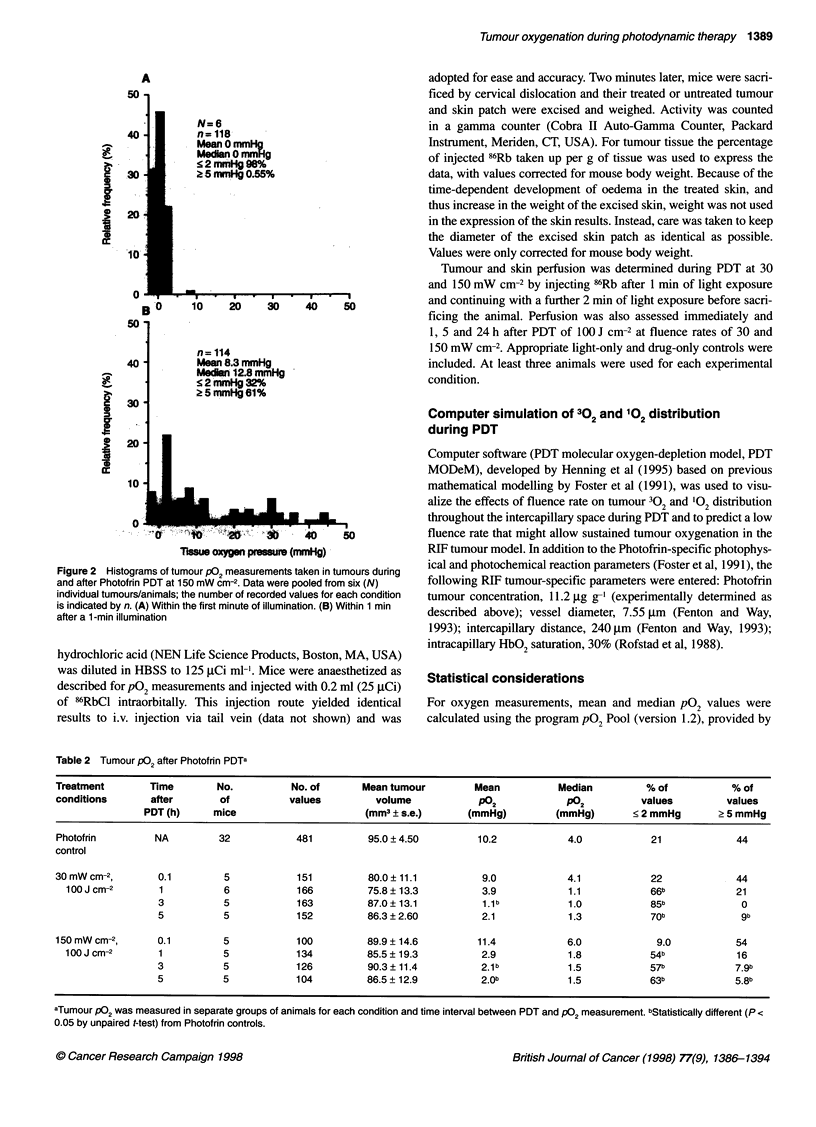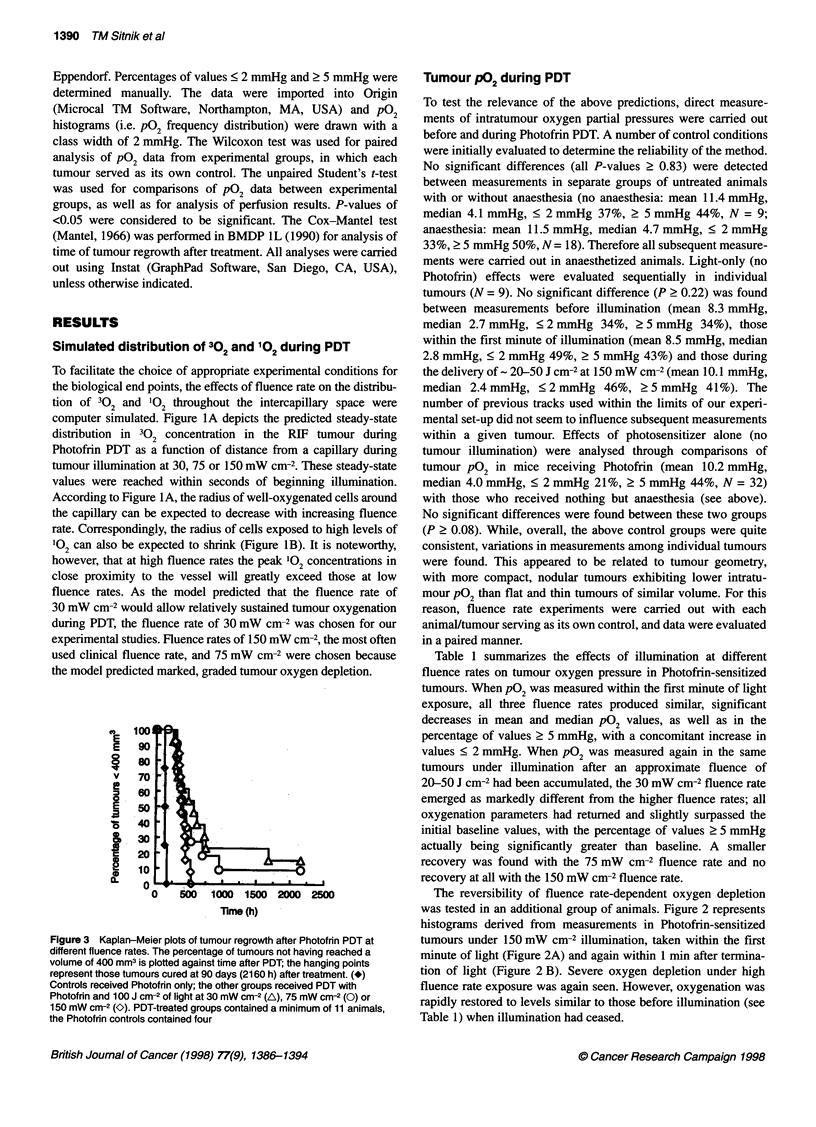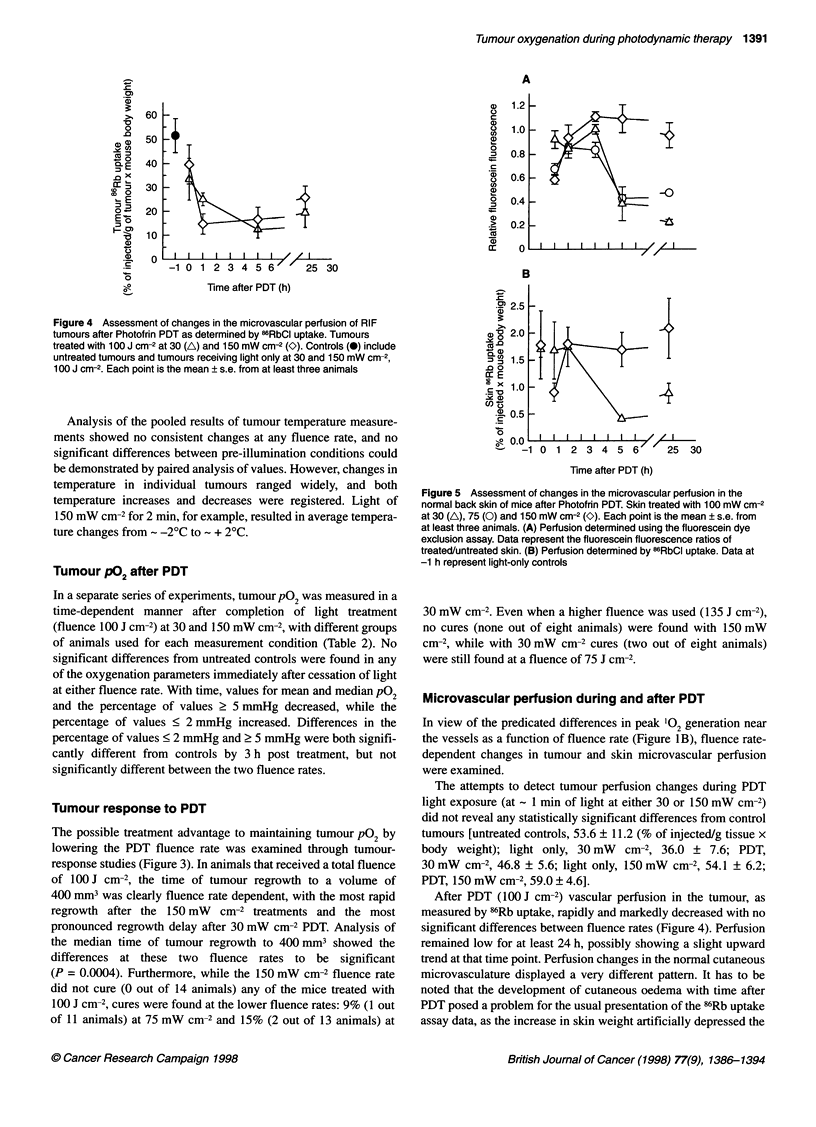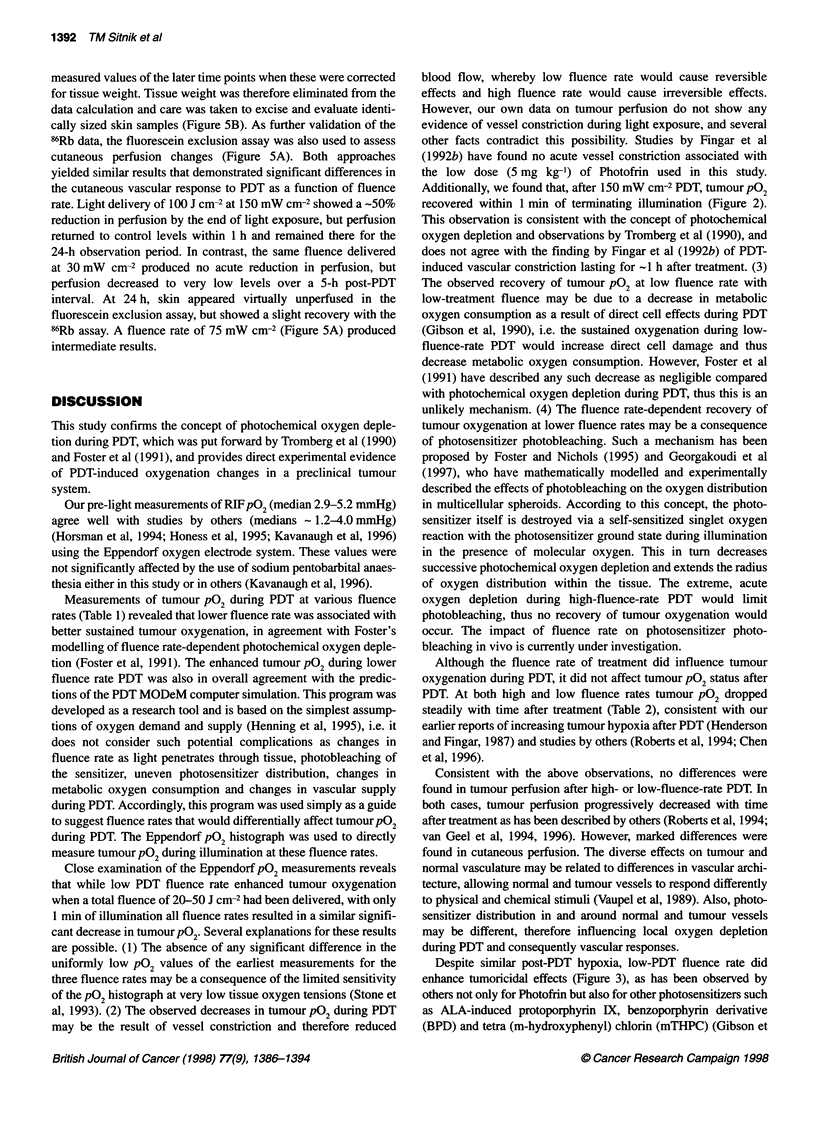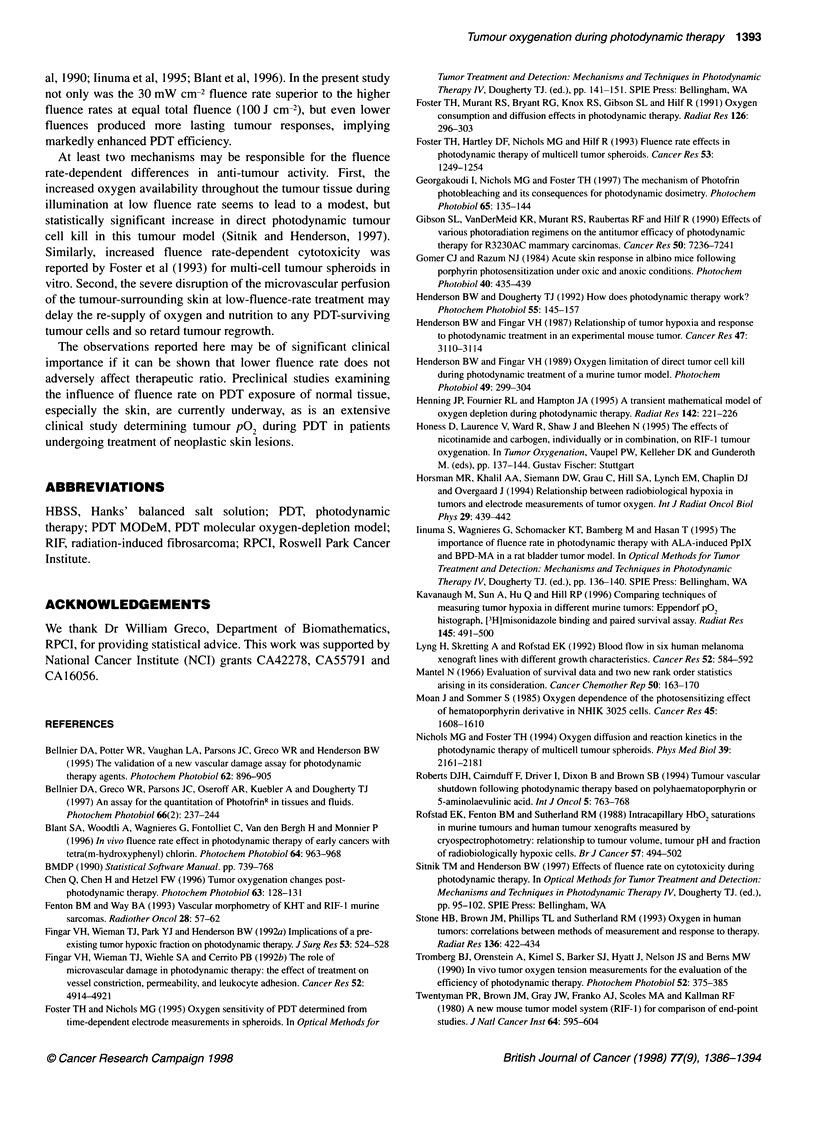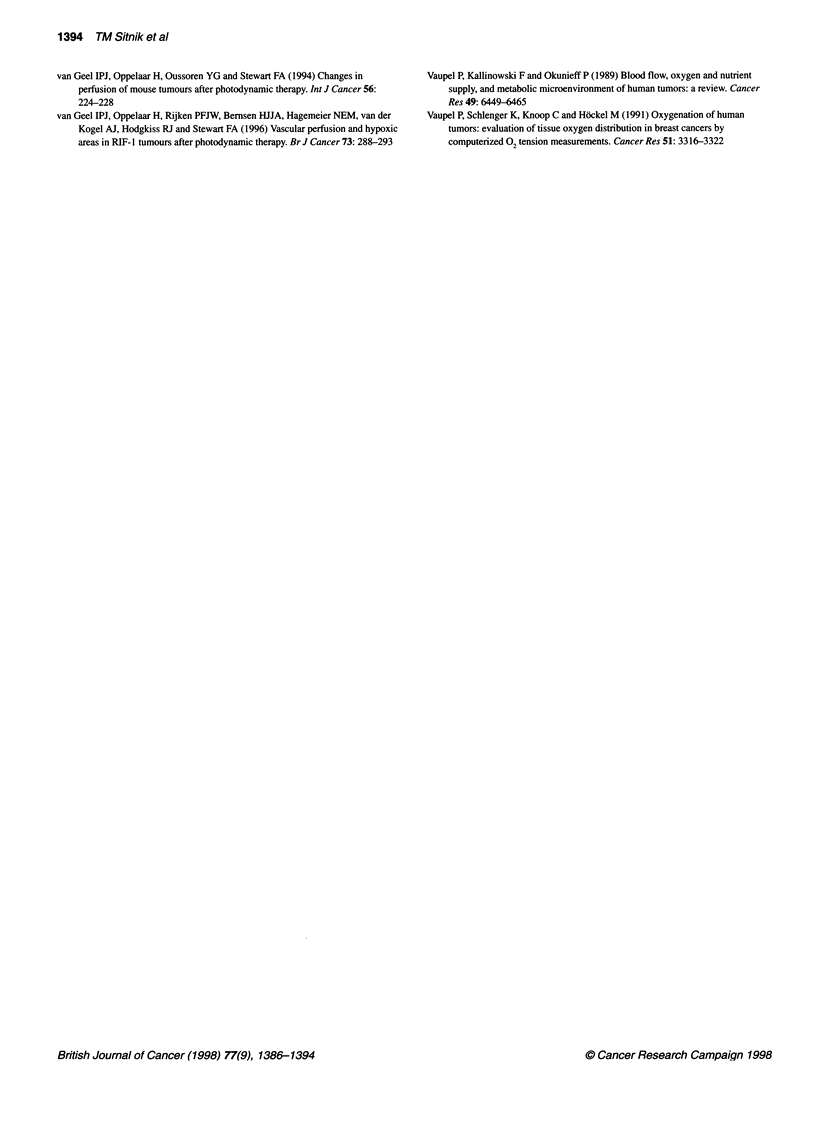# Reduction of tumour oxygenation during and after photodynamic therapy in vivo: effects of fluence rate.

**DOI:** 10.1038/bjc.1998.231

**Published:** 1998-05

**Authors:** T. M. Sitnik, J. A. Hampton, B. W. Henderson

**Affiliations:** Department of Radiation Biology, Roswell Park Cancer Institute, Buffalo, NY 14263, USA.

## Abstract

It has been proposed that the generation of O2 during photodynamic therapy (PDT) may lead to photochemical depletion of ambient tumour oxygen, thus causing acute hypoxia and limiting treatment effectiveness. We have studied the effects of fluence rate on pO2, in the murine RIF tumour during and after PDT using 5 mg kg(-1) Photofrin and fluence rates of 30, 75 or 150 mW cm(-2). Median pO2 before PDT ranged from 2.9 to 5.2 mmHg in three treatment groups. Within the first minute of illumination, median tumour pO2 decreased with all fluence rates to values between 0.7 and 1.1 mmHg. These effects were rapidly and completely reversible if illumination was interrupted. During prolonged illumination (20-50 J cm(-2)) pO2 recovered at the 30 mW cm(-2) fluence rate to a median value of 7.4 mmHg, but remained low at the 150 mW cm(-2) fluence rate (median pO2 1.7 mmHg). Fluence rate effects were not found after PDT, and at both 30 and 150 mW cm(-2) median tumour pO2 fell from control levels to 1.0-1.8 mmHg within 1-3 h after treatment conclusion. PDT with 100 J cm(-2) at 30 mW cm(-2) caused significantly (P = 0.0004) longer median tumour regrowth times than PDT at 150 mW cm(-2), indicating that lower fluence rate can improve PDT response. Vascular perfusion studies uncovered significant fluence rate-dependent differences in the responses of the normal and tumour vasculature. These data establish a direct relationship between tumour pO2, the fluence rate applied during PDT and treatment outcome. The findings are of immediate clinical relevance.


British Journal of Cancer (1998) 77(9), 1386–1394
© 1998 Cancer Research Campaign

### Reduction of tumour oxygenation during and after photodynamic therapy in vivo: effects of fluence rate

**TM Sitnik[1], JA Hampton[2] and BW Henderson[1]**

[1]Department of Radiation Biology, Roswell Park Cancer Institute (RPCI), Buffalo, 14263, NY, USA; [2]Departments of Pathology and Urology, Medical College of Ohio, Toledo, 43606, OH, USA

**Summary** It has been proposed that the generation of $^1O_2$ during photodynamic therapy (PDT) may lead to photochemical depletion of ambient tumour oxygen, thus causing acute hypoxia and limiting treatment effectiveness. We have studied the effects of fluence rate on $pO_2$, in the murine RIF tumour during and after PDT using 5 mg kg$^{-1}$ Photofrin and fluence rates of 30, 75 or 150 mW cm$^{-2}$. Median $pO_2$ before PDT ranged from 2.9 to 5.2 mmHg in three treatment groups. Within the first minute of illumination, median tumour $pO_2$ decreased with all fluence rates to values between 0.7 and 1.1 mmHg. These effects were rapidly and completely reversible if illumination was interrupted. During prolonged illumination (20–50 J cm$^{-2}$) $pO_2$ recovered at the 30 mW cm$^{-2}$ fluence rate to a median value of 7.4 mmHg, but remained low at the 150 mW cm$^{-2}$ fluence rate (median $pO_2$ 1.7 mmHg). Fluence rate effects were not found after PDT, and at both 30 and 150 mW cm$^{-2}$ median tumour $pO_2$ fell from control levels to 1.0–1.8 mmHg within 1–3 h after treatment conclusion. PDT with 100 J cm$^{-2}$ at 30 mW cm$^{-2}$ caused significantly ($P = 0.0004$) longer median tumour regrowth times than PDT at 150 mW cm$^{-2}$, indicating that lower fluence rate can improve PDT response. Vascular perfusion studies uncovered significant fluence rate-dependent differences in the responses of the normal and tumour vasculature. These data establish a direct relationship between tumour $pO_2$, the fluence rate applied during PDT and treatment outcome. The findings are of immediate clinical relevance.

**Keywords:** photodynamic therapy; fluence rate; tumour oxygenation; photochemical oxygen depletion; acute hypoxia; microvascular perfusion

Photodynamic therapy (PDT) with Photofrin is a recently approved modality for the treatment of cancer of the oesophagus, lung, bladder, stomach and cervix in various countries and is being tested clinically for the treatment of other malignancies and some benign conditions. In PDT, a tissue-localized photosensitizer is excited by visible light in the red or near-infrared spectral region. Cellular damage occurs when the photosensitizer in its excited triplet state transfers its energy to ground-state molecular oxygen ($^3O_2$), creating cytotoxic oxygen species, primarily singlet oxygen ($^1O_2$) (Henderson and Dougherty, 1992). It follows that $^3O_2$ is essential for PDT cytotoxicity. Indeed, it has been demonstrated in vitro and in vivo that cell and/or tissue damage are absent when PDT is given under anoxic conditions (Gomer and Razum, 1984; Moan and Sommer, 1985; Henderson and Fingar, 1987). For in vivo PDT effectiveness, tissue $pO_2$ may therefore be a determining factor.

In tumours, pre-existing regional hypoxia has been recognized as a barrier to effective treatment with other oxygen-dependent modalities, such as radiotherapy, and it may play a role in PDT as well (Fingar et al, 1992*a*). In addition, there exist two mechanisms by which PDT itself may induce tissue hypoxia and thus become self-limiting. Hypoxia may result from PDT-mediated damage to the microvasculature, which usually develops with time after PDT, but which can occur acutely during light delivery at high photosensitizer and light doses with certain photosensitizers (Henderson and Fingar, 1989). The second mechanism that may lead to tissue hypoxia is the consumption of $^3O_2$ in the PDT-mediated formation and consumption of $^1O_2$. Very rapid and substantial reductions in tissue oxygen tensions upon illumination of photosensitized tissue have been detected with transcutaneous oxygen electrodes by Tromberg et al (1990). Mathematical modelling by Foster et al (1991) has demonstrated that the rate of oxygen consumption during Photofrin PDT may be significant enough to move a fraction of the tumour into very low levels of oxygenation, outpacing the rate of oxygen diffusion from the capillaries and shrinking the radius of oxygenated tissue volume around them.

The rate of $^1O_2$ generation is influenced by the fluence rate of illumination, suggesting that $^3O_2$ consumption may in part be controlled by this treatment parameter. Studies in multicell tumour spheroids in vitro have revealed a strong relationship between the fluence rate, oxygen consumption (measured both by oxygen microelectrodes and modelled mathematically) and the fraction of PDT surviving cells (Foster et al, 1993; Nichols and Foster, 1994). The superior effectiveness of lower fluence rates in delaying tumour regrowth compared with treatments at higher fluence rates with the same overall fluence has been demonstrated by Gibson et al (1990) and Foster et al (1991). These results may be explained, at least theoretically, by more sustained tumour oxygenation throughout the course of treatment (Foster et al, 1991, 1993; Nichols and Foster, 1994).

This paper provides an experimental analysis of the relationships between fluence rate of light treatment, tumour oxygenation, vascular perfusion of tumour and normal skin, and tumour response.

*Received 30 April 1997*
*Revised 23 July 1997*
*Accepted 28 July 1997*

*Correspondence to:* BW Henderson

### MATERIALS AND METHODS

### Animals and tumour system

The RIF (radiation-induced fibrosarcoma) transplantable tumour, propagated in 8- to 12-week-old female $C_3Hf$/HeRos mice (obtained from the RPCI breeding facilities), was used for all experiments requiring tumours. All aspects of animal experimentation and husbandry were carried out in compliance with federal and state standards, and were approved by the Animal Care and Use Committee of RPCI. For tumour inoculation, $3 \times 10^5$ RIF cells, obtained from established tumours by enzyme digestion according to established procedures of successive in vitro and in vivo passage (Twentyman et al, 1980), were injected intradermally on the animals' right shoulder, the inoculation site having been shaved and depilated with Nair 24 h earlier. Animals were used 5–7 days after inoculation, when their tumours were about 4–6 mm in diameter and 2–4 mm in height. For microvascular perfusion studies of the skin, an area (1 cm in diameter) was also depilated on the left shoulder.

### Photosensitizers

Photofrin (QLT PhotoTherapeutics, Vancouver, BC, Canada) was used in all experiments. It was reconstituted in 5% dextrose in water (D5W) according to company instructions. Mice were injected i.v. (via tail vein) with 5 mg $kg^{-1}$ Photofrin 24 h before light exposure.

### Determination of photosensitizer tumour concentrations

Tumour concentrations of Photofrin were determined 24 h after photosensitizer administration by fluorescence measurements after tissue solubilization as described (Bellnier et al, 1997). Fluorescence spectra of test samples were compared with a standard curve and μg of drug per g of tumour was calculated. The obtained values were used as input parameters for the computer simulation of $^3O_2$ and $^1O_2$ distributions (see below).

### In vivo photodynamic treatment

Twenty-four hours after photosensitizer administration, tumours and/or normal skin were exposed to light from a dye laser (Model 375, Spectra-Physics, Mountain View, CA, USA) pumped by an argon-ion laser (either Trimedyne Optilase, Santa Anna, CA, USA, or Model 171, Spectra-Physics). The dye laser was coupled to a 200-μm diameter quartz fibre fitted with a GRIN lens to provide uniform illumination. The light was set at a wavelength of 630 nm with a monochromator (DMC1-02, Optometrics USA, Ayer, MA, USA). Power output was measured with a thermal disc calorimeter (Model 210, Coherent, Auburn, CA, USA), and the power density was adjusted over a treatment field of 1 cm in diameter to yield fluence rates of 30, 75 or 150 mW $cm^{-2}$. Total treatment fluences varied with the experimental end point to be measured and are given under Results.

### Measurement of tumour $pO_2$

Intratumour $pO_2$ was measured polarographically using the Eppendorf $pO_2$ Histograph (Eppendorf Scientific, Madison, WI, USA) (Vaupel et al, 1991). Before and between measurements, the instrument was calibrated in 0.9% saline bubbled alternately with air and nitrogen to set 100% and 0% $pO_2$ currents. Ambient air pressure and tumour temperature (measured using an Omega 30 gauge, $^1/_2$-inch needle thermocouple) were recorded and used to post-calibrate the data. For each experimental condition, the last track used for $pO_2$ measurement was used for insertion of the thermocouple.

Tumours ranging in size from 45 to 145 $mm^3$ were used for oxygen measurements; at that size, no spontaneous tumour necrosis is evident. Measurements were performed under sodium pentobarbital anaesthesia (66 mg $kg^{-1}$ i.p.) unless otherwise indicated. The 300-μm-diameter polarographic needle probe was aligned at the tumour surface after creation of a pinpoint hole, using a hypodermic needle, in the skin covering the tumour. The probe was advanced one step to ensure the tip was within the tumour, and automatic probe advancement was begun after the $pO_2$ values had stabilized. Probe advancement was set to a 0.7-mm forward step and a 0.3-mm retraction step for each reading. Probe track length and number of tracks measured were determined by tumour dimensions. Tracking was horizontal through the tumour, and care was taken to choose comparable track positions (tumour periphery vs centre) for control and experimental measurements. One to two tracks were measured for each measurement condition (see below), with a maximum of six tracks per tumour.

PDT treatments were carried out at fluence rates of 30, 75 or 150 mW $cm^{-2}$. The following measurement protocols were used: (1) in each tumour, $pO_2$ was measured immediately before illumination, within the first minute into illumination, and again during illumination when similar total light fluences in the range of 20–50 J $cm^{-2}$ were reached; for measurements within the first

**Figure 1** Simulated fluence rate-dependent PDT effects on RIF tumour oxygenation, plotted with PDT MODeM as a function of radial distance from a capillary. **(A)** $^3O_2$ distribution in μM. **(B)** $^1O_2$ distribution in pM. ●, Metabolic $^3O_2$ consumption without PDT; PDT at 30 (△), 75 (○) or 150 mW $cm^{-2}$ (◇)

minute the probe was aligned before illumination, while for measurements at the longer time intervals the probe was aligned during illumination; the fluence range of 20–50 J $cm^{-2}$ was dictated by the time required to align the probe and carry out the $pO_2$ measurements, i.e. measurements at 150 mW $cm^{-2}$ were carried out between 2 and 6 min into illumination, whereas measurements at 30 mW $cm^{-2}$ were carried out between 20 and 26 min into illumination; (2) in each tumour, $pO_2$ was measured within the first minute of illumination, the light was removed and $pO_2$ was again measured within the first minute after illumination; (3) in separate groups of tumours, $pO_2$ was measured immediately and at 1, 3 and 5 h after a full treatment dose (100 J $cm^{-2}$) of PDT. Appropriate control measurements were also performed on totally untreated tumours and tumours receiving light only, as well as on untreated tumours in mice with or without anaesthesia. In experiments that required prolonged illumination, mice were kept warm using a microwaveable hot pad. Five to ten animals were used per experimental group. Data are expressed as mean and median values, as well as percentages of values ≤ 2 mmHg and ≥ 5 mmHg. These parameters were chosen for analysis because they lie within the range of oxygen concentrations reported in the literature, in which $^1O_2$ formation becomes limited by oxygen availability (Moan and Sommer, 1985; Foster and Nichols, 1995).

### Determination of tumour response

The animals were restrained, unanaesthetized, in specially designed holders and PDT was carried out at fluence rates of 30, 75 and 150 mW $cm^{-2}$, with a total fluence of 75, 100 and 135 J $cm^{-2}$ being delivered at each fluence rate. After treatment, tumour size was recorded 6 days a week for 30 days, and then weekly up to 90 days. Tumour size was measured in two orthogonal dimensions using an electronic calliper (Ultra-Cal Mark III, Fred V Fowler, Newton, MA, USA) and automatically entered into a spreadsheet (Excel, Microsoft, Redmond, WA, USA). Tumour volume was calculated with the formula $V = (l \times w^2)/2$, and time to reach 400 $mm^3$ was estimated using the natural log of the volume. Groups of eight to thirteen animals were evaluated. Results are presented as Kaplan–Meier curves in which the percentage of animals with tumour volumes less than 400 $mm^3$ is plotted against the number of hours since treatment (GraphPad Prism, GraphPad Software, San Diego, CA, USA).

### Fluorescein exclusion assay of skin microvascular perfusion

This method for assessing microvascular perfusion of the normal skin before and after PDT has been described in detail earlier (Bellnier et al, 1995). Briefly, immediately after PDT light exposure and at 1, 3, 5 and 24 h thereafter, unanaesthetized animals were injected with 0.2 ml of 2 mg $ml^{-1}$ fluorescein (JT Baker Chemical, Phillipsburg, NJ, USA) in Hanks' balanced salt solution (HBSS) via the orbital plexus. Five to 8 min after fluorescein injection, fluorescein-specific fluorescence within and outside the treatment field (four readings each) was detected by a fluorometer. At least three mice were evaluated for each data point. Data were recorded as the ratio (treated/untreated sites) of the mean values of the measurements. The PDT treatment parameters for these experiments were a total fluence of 100 J $cm^{-2}$ at fluence rates of 30, 75 and 100 mW $cm^{-2}$.

### $^{86}$RbCl uptake as a measure of tumour and skin microvascular perfusion

This technique has been described by others (Lyng et al, 1992) and was used with slight modifications. Briefly, $^{86}$RbCl in 0.5 M

**Table 1** Effects of fluence rate on tumour $pO_2$ during Photofrin PDT[a]

| Treatment condition | No. of mice | Mean tumour volume ($mm^3$ ± s.e.) | No. of values | Mean $pO_2$ (mmHg) | Median $pO_2$ (mmHg) | % of values ≤ 2 mmHg | % of values ≥ 5 mmHg |
|---|---|---|---|---|---|---|---|
| *30 mW $cm^{-2}$* | | | | | | | |
| Before illumination | 10 | 90.5 ± 8.1 | 188 | 9.0 | 2.9 | 28 | 36 |
| Within 1 min | | | 186 | 1.7 (*P* = 0.002) | 1.1 (*P* = 0.002) | 76 (*P* = 0.004) | 9.1 (*P* = 0.004) |
| 20–50 J $cm^{-2}$ (20–26 min) | | | 191 | 14.6 (*P* = 0.7) | 7.4 (*P* = 0.4) | 8.4 (*P* = 0.3) | 61 (*P* = 0.02) |
| *75 mW $cm^{-2}$* | | | | | | | |
| Before illumination | 8 | 103.6 ± 7.2 | 123 | 8.3 | 3.9 | 22 | 38 |
| Within 1 min | | | 112 | 0.9 (*P* = 0.008) | 0.7 (*P* = 0.02) | 91 (*P* = 0.02) | 1.8 (*P* = 0.02) |
| 20–50 J $cm^{-2}$ (6–10 min) | | | 94 | 4.0 (*P* = 0.02) | 2.8 (*P* = 0.1) | 37 (*P* = 0.4) | 18 (*P* = 0.03) |
| *150 mW $cm^{-2}$* | | | | | | | |
| Before illumination | 10 | 92.9 ± 8.9 | 127 | 10.3 | 5.2 | 17 | 53 |
| Within 1 min | | | 129 | 1.7 (*P* = 0.002) | 0.7 (*P* = 0.002) | 73 (*P* = 0.004) | 7 (*P* = 0.002) |
| 20–50 J $cm^{-2}$ (2–6 min) | | | 165 | 2.1 (*P* = 0.002) | 1.7 (*P* = 0.004) | 61 (*P* = 0.01) | 8.5 (*P* = 0.002) |

[a]Tumour $pO_2$ was measured in three groups of animals, one for each fluence rate; changes in $pO_2$ were recorded for individual tumours from pre-illumination through illumination as described; data were calculated from the pooled results of all measurements for each condition; *P*-values were calculated for differences between the pre-illumination values and values of a given illumination condition using a paired test comparing changes within individual tumours.

 © Cancer Research Campaign 1998

**Figure 2** Histograms of tumour $pO_2$ measurements taken in tumours during and after Photofrin PDT at 150 mW $cm^{-2}$. Data were pooled from six (*N*) individual tumours/animals; the number of recorded values for each condition is indicated by *n*. (**A**) Within the first minute of illumination. (**B**) Within 1 min after a 1-min illumination hydrochloric acid (NEN Life Science Products, Boston, MA, USA) was diluted in HBSS to 125 μCi $ml^{-1}$. Mice were anaesthetized as described for $pO_2$ measurements and injected with 0.2 ml (25 μCi) of $^{86}RbCl$ intraorbitally. This injection route yielded identical results to i.v. injection via tail vein (data not shown) and was adopted for ease and accuracy. Two minutes later, mice were sacrificed by cervical dislocation and their treated or untreated tumour and skin patch were excised and weighed. Activity was counted in a gamma counter (Cobra II Auto-Gamma Counter, Packard Instrument, Meriden, CT, USA). For tumour tissue the percentage of injected $^{86}Rb$ taken up per g of tissue was used to express the data, with values corrected for mouse body weight. Because of the time-dependent development of oedema in the treated skin, and thus increase in the weight of the excised skin, weight was not used in the expression of the skin results. Instead, care was taken to keep the diameter of the excised skin patch as identical as possible. Values were only corrected for mouse body weight.

Tumour and skin perfusion was determined during PDT at 30 and 150 mW $cm^{-2}$ by injecting $^{86}Rb$ after 1 min of light exposure and continuing with a further 2 min of light exposure before sacrificing the animal. Perfusion was also assessed immediately and 1, 5 and 24 h after PDT of 100 J $cm^{-2}$ at fluence rates of 30 and 150 mW $cm^{-2}$. Appropriate light-only and drug-only controls were included. At least three animals were used for each experimental condition.

### Computer simulation of $^3O_2$ and $^1O_2$ distribution during PDT

Computer software (PDT molecular oxygen-depletion model, PDT MODeM), developed by Henning et al (1995) based on previous mathematical modelling by Foster et al (1991), was used to visualize the effects of fluence rate on tumour $^3O_2$ and $^1O_2$ distribution throughout the intercapillary space during PDT and to predict a low fluence rate that might allow sustained tumour oxygenation in the RIF tumour model. In addition to the Photofrin-specific photophysical and photochemical reaction parameters (Foster et al, 1991), the following RIF tumour-specific parameters were entered: Photofrin tumour concentration, 11.2 μg $g^{-1}$ (experimentally determined as described above); vessel diameter, 7.55 μm (Fenton and Way, 1993); intercapillary distance, 240 μm (Fenton and Way, 1993); intracapillary $HbO_2$ saturation, 30% (Rofstad et al, 1988).

### Statistical considerations

For oxygen measurements, mean and median $pO_2$ values were calculated using the program $pO_2$ Pool (version 1.2), provided by

**Table 2** Tumour $pO_2$ after Photofrin PDT[a]

| Treatment conditions | Time after PDT (h) | No. of mice | No. of values | Mean tumour volume ($mm^3$ ± s.e.) | Mean $pO_2$ (mmHg) | Median $pO_2$ (mmHg) | % of values ≤ 2 mmHg | % of values ≥ 5 mmHg |
|---|---|---|---|---|---|---|---|---|
| Photofrin control | NA | 32 | 481 | 95.0 ± 4.50 | 10.2 | 4.0 | 21 | 44 |
| 30 mW $cm^{-2}$, 100 J $cm^{-2}$ | 0.1 | 5 | 151 | 80.0 ± 11.1 | 9.0 | 4.1 | 22 | 44 |
| | 1 | 6 | 166 | 75.8 ± 13.3 | 3.9 | 1.1 | 66[b] | 21 |
| | 3 | 5 | 163 | 87.0 ± 13.1 | 1.1[b] | 1.0 | 85[b] | 0 |
| | 5 | 5 | 152 | 86.3 ± 2.60 | 2.1 | 1.3 | 70[b] | 9[b] |
| 150 mW $cm^{-2}$, 100 J $cm^{-2}$ | 0.1 | 5 | 100 | 89.9 ± 14.6 | 11.4 | 6.0 | 9.0 | 54 |
| | 1 | 5 | 134 | 85.5 ± 19.3 | 2.9 | 1.8 | 54[b] | 16 |
| | 3 | 5 | 126 | 90.3 ± 11.4 | 2.1[b] | 1.5 | 57[b] | 7.9[b] |
| | 5 | 5 | 104 | 86.5 ± 12.9 | 2.0[b] | 1.5 | 63[b] | 5.8[b] |

[a]Tumour $pO_2$ was measured in separate groups of animals for each condition and time interval between PDT and $pO_2$ measurement. [b]Statistically different ($P < 0.05$ by unpaired *t*-test) from Photofrin controls.

© Cancer Research Campaign 1998

Eppendorf. Percentages of values ≤ 2 mmHg and ≥ 5 mmHg were determined manually. The data were imported into Origin (Microcal TM Software, Northampton, MA, USA) and $pO_2$ histograms (i.e. $pO_2$ frequency distribution) were drawn with a class width of 2 mmHg. The Wilcoxon test was used for paired analysis of $pO_2$ data from experimental groups, in which each tumour served as its own control. The unpaired Student's *t*-test was used for comparisons of $pO_2$ data between experimental groups, as well as for analysis of perfusion results. *P*-values of <0.05 were considered to be significant. The Cox–Mantel test (Mantel, 1966) was performed in BMDP 1L (1990) for analysis of time of tumour regrowth after treatment. All analyses were carried out using Instat (GraphPad Software, San Diego, CA, USA), unless otherwise indicated.

### RESULTS

### Simulated distribution of $^3O_2$ and $^1O_2$ during PDT

To facilitate the choice of appropriate experimental conditions for the biological end points, the effects of fluence rate on the distribution of $^3O_2$ and $^1O_2$ throughout the intercapillary space were computer simulated. Figure 1A depicts the predicted steady-state distribution in $^3O_2$ concentration in the RIF tumour during Photofrin PDT as a function of distance from a capillary during tumour illumination at 30, 75 or 150 mW cm$^{-2}$. These steady-state values were reached within seconds of beginning illumination. According to Figure 1A, the radius of well-oxygenated cells around the capillary can be expected to decrease with increasing fluence rate. Correspondingly, the radius of cells exposed to high levels of $^1O_2$ can also be expected to shrink (Figure 1B). It is noteworthy, however, that at high fluence rates the peak $^1O_2$ concentrations in close proximity to the vessel will greatly exceed those at low fluence rates. As the model predicted that the fluence rate of 30 mW cm$^{-2}$ would allow relatively sustained tumour oxygenation during PDT, the fluence rate of 30 mW cm$^{-2}$ was chosen for our experimental studies. Fluence rates of 150 mW cm$^{-2}$, the most often used clinical fluence rate, and 75 mW cm$^{-2}$ were chosen because the model predicted marked, graded tumour oxygen depletion.

**Figure 3** Kaplan–Meier plots of tumour regrowth after Photofrin PDT at different fluence rates. The percentage of tumours not having reached a volume of 400 mm$^3$ is plotted against time after PDT; the hanging points represent those tumours cured at 90 days (2160 h) after treatment. (◆) Controls received Photofrin only; the other groups received PDT with Photofrin and 100 J cm$^{-2}$ of light at 30 mW cm$^{-2}$ (△), 75 mW cm$^{-2}$ (○) or 150 mW cm$^{-2}$ (◇). PDT-treated groups contained a minimum of 11 animals, the Photofrin controls contained four

### Tumour $pO_2$ during PDT

To test the relevance of the above predictions, direct measurements of intratumour oxygen partial pressures were carried out before and during Photofrin PDT. A number of control conditions were initially evaluated to determine the reliability of the method. No significant differences (all *P*-values ≥ 0.83) were detected between measurements in separate groups of untreated animals with or without anaesthesia (no anaesthesia: mean 11.4 mmHg, median 4.1 mmHg, ≤ 2 mmHg 37%, ≥ 5 mmHg 44%, *N* = 9; anaesthesia: mean 11.5 mmHg, median 4.7 mmHg, ≤ 2 mmHg 33%, ≥ 5 mmHg 50%, *N* = 18). Therefore all subsequent measurements were carried out in anaesthetized animals. Light-only (no Photofrin) effects were evaluated sequentially in individual tumours (*N* = 9). No significant difference (*P* ≥ 0.22) was found between measurements before illumination (mean 8.3 mmHg, median 2.7 mmHg, ≤ 2 mmHg 34%, ≥ 5 mmHg 34%), those within the first minute of illumination (mean 8.5 mmHg, median 2.8 mmHg, ≤ 2 mmHg 49%, ≥ 5 mmHg 43%) and those during the delivery of ~ 20–50 J cm$^{-2}$ at 150 mW cm$^{-2}$ (mean 10.1 mmHg, median 2.4 mmHg, ≤ 2 mmHg 46%, ≥ 5 mmHg 41%). The number of previous tracks used within the limits of our experimental set-up did not seem to influence subsequent measurements within a given tumour. Effects of photosensitizer alone (no tumour illumination) were analysed through comparisons of tumour $pO_2$ in mice receiving Photofrin (mean 10.2 mmHg, median 4.0 mmHg, ≤ 2 mmHg 21%, ≥ 5 mmHg 44%, *N* = 32) with those who received nothing but anaesthesia (see above). No significant differences were found between these two groups (*P* ≥ 0.08). While, overall, the above control groups were quite consistent, variations in measurements among individual tumours were found. This appeared to be related to tumour geometry, with more compact, nodular tumours exhibiting lower intratumour $pO_2$ than flat and thin tumours of similar volume. For this reason, fluence rate experiments were carried out with each animal/tumour serving as its own control, and data were evaluated in a paired manner.

Table 1 summarizes the effects of illumination at different fluence rates on tumour oxygen pressure in Photofrin-sensitized tumours. When $pO_2$ was measured within the first minute of light exposure, all three fluence rates produced similar, significant decreases in mean and median $pO_2$ values, as well as in the percentage of values ≥ 5 mmHg, with a concomitant increase in values ≤ 2 mmHg. When $pO_2$ was measured again in the same tumours under illumination after an approximate fluence of 20–50 J cm$^{-2}$ had been accumulated, the 30 mW cm$^{-2}$ fluence rate emerged as markedly different from the higher fluence rates; all oxygenation parameters had returned and slightly surpassed the initial baseline values, with the percentage of values ≥ 5 mmHg actually being significantly greater than baseline. A smaller recovery was found with the 75 mW cm$^{-2}$ fluence rate and no recovery at all with the 150 mW cm$^{-2}$ fluence rate.

The reversibility of fluence rate-dependent oxygen depletion was tested in an additional group of animals. Figure 2 represents histograms derived from measurements in Photofrin-sensitized tumours under 150 mW cm$^{-2}$ illumination, taken within the first minute of light (Figure 2A) and again within 1 min after termination of light (Figure 2 B). Severe oxygen depletion under high fluence rate exposure was again seen. However, oxygenation was rapidly restored to levels similar to those before illumination (see Table 1) when illumination had ceased.

© Cancer Research Campaign 1998

**Figure 4** Assessment of changes in the microvascular perfusion of RIF tumours after Photofrin PDT as determined by $^{86}$RbCl uptake. Tumours treated with 100 J cm$^{-2}$ at 30 (△) and 150 mW cm$^{-2}$ (◇). Controls (●) include untreated tumours and tumours receiving light only at 30 and 150 mW cm$^{-2}$, 100 J cm$^{-2}$. Each point is the mean ± s.e. from at least three animals

Analysis of the pooled results of tumour temperature measurements showed no consistent changes at any fluence rate, and no significant differences between pre-illumination conditions could be demonstrated by paired analysis of values. However, changes in temperature in individual tumours ranged widely, and both temperature increases and decreases were registered. Light of 150 mW cm$^{-2}$ for 2 min, for example, resulted in average temperature changes from ~ –2°C to ~ + 2°C.

### Tumour $pO_2$ after PDT

In a separate series of experiments, tumour $pO_2$ was measured in a time-dependent manner after completion of light treatment (fluence 100 J cm$^{-2}$) at 30 and 150 mW cm$^{-2}$, with different groups of animals used for each measurement condition (Table 2). No significant differences from untreated controls were found in any of the oxygenation parameters immediately after cessation of light at either fluence rate. With time, values for mean and median $pO_2$ and the percentage of values ≥ 5 mmHg decreased, while the percentage of values ≤ 2 mmHg increased. Differences in the percentage of values ≤ 2 mmHg and ≥ 5 mmHg were both significantly different from controls by 3 h post treatment, but not significantly different between the two fluence rates.

### Tumour response to PDT

The possible treatment advantage to maintaining tumour $pO_2$ by lowering the PDT fluence rate was examined through tumour-response studies (Figure 3). In animals that received a total fluence of 100 J cm$^{-2}$, the time of tumour regrowth to a volume of 400 mm$^3$ was clearly fluence rate dependent, with the most rapid regrowth after the 150 mW cm$^{-2}$ treatments and the most pronounced regrowth delay after 30 mW cm$^{-2}$ PDT. Analysis of the median time of tumour regrowth to 400 mm$^3$ showed the differences at these two fluence rates to be significant ($P$ = 0.0004). Furthermore, while the 150 mW cm$^{-2}$ fluence rate did not cure (0 out of 14 animals) any of the mice treated with 100 J cm$^{-2}$, cures were found at the lower fluence rates: 9% (1 out of 11 animals) at 75 mW cm$^{-2}$ and 15% (2 out of 13 animals) at

**Figure 5** Assessment of changes in the microvascular perfusion in the normal back skin of mice after Photofrin PDT. Skin treated with 100 mW cm$^{-2}$ at 30 (△), 75 (○) and 150 mW cm$^{-2}$ (◇). Each point is the mean ± s.e. from at least three animals. (**A**) Perfusion determined using the fluorescein dye exclusion assay. Data represent the fluorescein fluorescence ratios of treated/untreated skin. (**B**) Perfusion determined by $^{86}$RbCl uptake. Data at –1 h represent light-only controls

30 mW cm$^{-2}$. Even when a higher fluence was used (135 J cm$^{-2}$), no cures (none out of eight animals) were found with 150 mW cm$^{-2}$, while with 30 mW cm$^{-2}$ cures (two out of eight animals) were still found at a fluence of 75 J cm$^{-2}$.

### Microvascular perfusion during and after PDT

In view of the predicated differences in peak $^1O_2$ generation near the vessels as a function of fluence rate (Figure 1B), fluence rate-dependent changes in tumour and skin microvascular perfusion were examined.

The attempts to detect tumour perfusion changes during PDT light exposure (at ~ 1 min of light at either 30 or 150 mW cm$^{-2}$) did not reveal any statistically significant differences from control tumours [untreated controls, 53.6 ± 11.2 (% of injected/g tissue × body weight); light only, 30 mW cm$^{-2}$, 36.0 ± 7.6; PDT, 30 mW cm$^{-2}$, 46.8 ± 5.6; light only, 150 mW cm$^{-2}$, 54.1 ± 6.2; PDT, 150 mW cm$^{-2}$, 59.0 ± 4.6].

After PDT (100 J cm$^{-2}$) vascular perfusion in the tumour, as measured by $^{86}$Rb uptake, rapidly and markedly decreased with no significant differences between fluence rates (Figure 4). Perfusion remained low for at least 24 h, possibly showing a slight upward trend at that time point. Perfusion changes in the normal cutaneous microvasculature displayed a very different pattern. It has to be noted that the development of cutaneous oedema with time after PDT posed a problem for the usual presentation of the $^{86}$Rb uptake assay data, as the increase in skin weight artificially depressed the

© Cancer Research Campaign 1998

measured values of the later time points when these were corrected for tissue weight. Tissue weight was therefore eliminated from the data calculation and care was taken to excise and evaluate identically sized skin samples (Figure 5B). As further validation of the $^{86}$Rb data, the fluorescein exclusion assay was also used to assess cutaneous perfusion changes (Figure 5A). Both approaches yielded similar results that demonstrated significant differences in the cutaneous vascular response to PDT as a function of fluence rate. Light delivery of 100 J cm$^{-2}$ at 150 mW cm$^{-2}$ showed a ~50% reduction in perfusion by the end of light exposure, but perfusion returned to control levels within 1 h and remained there for the 24-h observation period. In contrast, the same fluence delivered at 30 mW cm$^{-2}$ produced no acute reduction in perfusion, but perfusion decreased to very low levels over a 5-h post-PDT interval. At 24 h, skin appeared virtually unperfused in the fluorescein exclusion assay, but showed a slight recovery with the $^{86}$Rb assay. A fluence rate of 75 mW cm$^{-2}$ (Figure 5A) produced intermediate results.

### DISCUSSION

This study confirms the concept of photochemical oxygen depletion during PDT, which was put forward by Tromberg et al (1990) and Foster et al (1991), and provides direct experimental evidence of PDT-induced oxygenation changes in a preclinical tumour system.

Our pre-light measurements of RIF $pO_2$ (median 2.9–5.2 mmHg) agree well with studies by others (medians ~ 1.2–4.0 mmHg) (Horsman et al, 1994; Honess et al, 1995; Kavanaugh et al, 1996) using the Eppendorf oxygen electrode system. These values were not significantly affected by the use of sodium pentobarbital anaesthesia either in this study or in others (Kavanaugh et al, 1996).

Measurements of tumour $pO_2$ during PDT at various fluence rates (Table 1) revealed that lower fluence rate was associated with better sustained tumour oxygenation, in agreement with Foster's modelling of fluence rate-dependent photochemical oxygen depletion (Foster et al, 1991). The enhanced tumour $pO_2$ during lower fluence rate PDT was also in overall agreement with the predictions of the PDT MODeM computer simulation. This program was developed as a research tool and is based on the simplest assumptions of oxygen demand and supply (Henning et al, 1995), i.e. it does not consider such potential complications as changes in fluence rate as light penetrates through tissue, photobleaching of the sensitizer, uneven photosensitizer distribution, changes in metabolic oxygen consumption and changes in vascular supply during PDT. Accordingly, this program was used simply as a guide to suggest fluence rates that would differentially affect tumour $pO_2$ during PDT. The Eppendorf $pO_2$ histograph was used to directly measure tumour $pO_2$ during illumination at these fluence rates.

Close examination of the Eppendorf $pO_2$ measurements reveals that while low PDT fluence rate enhanced tumour oxygenation when a total fluence of 20–50 J cm$^{-2}$ had been delivered, with only 1 min of illumination all fluence rates resulted in a similar significant decrease in tumour $pO_2$. Several explanations for these results are possible. (1) The absence of any significant difference in the uniformly low $pO_2$ values of the earliest measurements for the three fluence rates may be a consequence of the limited sensitivity of the $pO_2$ histograph at very low tissue oxygen tensions (Stone et al, 1993). (2) The observed decreases in tumour $pO_2$ during PDT may be the result of vessel constriction and therefore reduced blood flow, whereby low fluence rate would cause reversible effects and high fluence rate would cause irreversible effects. However, our own data on tumour perfusion do not show any evidence of vessel constriction during light exposure, and several other facts contradict this possibility. Studies by Fingar et al (1992*b*) have found no acute vessel constriction associated with the low dose (5 mg kg$^{-1}$) of Photofrin used in this study. Additionally, we found that, after 150 mW cm$^{-2}$ PDT, tumour $pO_2$ recovered within 1 min of terminating illumination (Figure 2). This observation is consistent with the concept of photochemical oxygen depletion and observations by Tromberg et al (1990), and does not agree with the finding by Fingar et al (1992*b*) of PDT-induced vascular constriction lasting for ~1 h after treatment. (3) The observed recovery of tumour $pO_2$ at low fluence rate with low-treatment fluence may be due to a decrease in metabolic oxygen consumption as a result of direct cell effects during PDT (Gibson et al, 1990), i.e. the sustained oxygenation during low-fluence-rate PDT would increase direct cell damage and thus decrease metabolic oxygen consumption. However, Foster et al (1991) have described any such decrease as negligible compared with photochemical oxygen depletion during PDT, thus this is an unlikely mechanism. (4) The fluence rate-dependent recovery of tumour oxygenation at lower fluence rates may be a consequence of photosensitizer photobleaching. Such a mechanism has been proposed by Foster and Nichols (1995) and Georgakoudi et al (1997), who have mathematically modelled and experimentally described the effects of photobleaching on the oxygen distribution in multicellular spheroids. According to this concept, the photosensitizer itself is destroyed via a self-sensitized singlet oxygen reaction with the photosensitizer ground state during illumination in the presence of molecular oxygen. This in turn decreases successive photochemical oxygen depletion and extends the radius of oxygen distribution within the tissue. The extreme, acute oxygen depletion during high-fluence-rate PDT would limit photobleaching, thus no recovery of tumour oxygenation would occur. The impact of fluence rate on photosensitizer photobleaching in vivo is currently under investigation.

Although the fluence rate of treatment did influence tumour oxygenation during PDT, it did not affect tumour $pO_2$ status after PDT. At both high and low fluence rates tumour $pO_2$ dropped steadily with time after treatment (Table 2), consistent with our earlier reports of increasing tumour hypoxia after PDT (Henderson and Fingar, 1987) and studies by others (Roberts et al, 1994; Chen et al, 1996).

Consistent with the above observations, no differences were found in tumour perfusion after high- or low-fluence-rate PDT. In both cases, tumour perfusion progressively decreased with time after treatment as has been described by others (Roberts et al, 1994; van Geel et al, 1994, 1996). However, marked differences were found in cutaneous perfusion. The diverse effects on tumour and normal vasculature may be related to differences in vascular architecture, allowing normal and tumour vessels to respond differently to physical and chemical stimuli (Vaupel et al, 1989). Also, photosensitizer distribution in and around normal and tumour vessels may be different, therefore influencing local oxygen depletion during PDT and consequently vascular responses.

Despite similar post-PDT hypoxia, low-PDT fluence rate did enhance tumoricidal effects (Figure 3), as has been observed by others not only for Photofrin but also for other photosensitizers such as ALA-induced protoporphyrin IX, benzoporphyrin derivative (BPD) and tetra (m-hydroxyphenyl) chlorin (mTHPC) (Gibson et

© Cancer Research Campaign 1998

al, 1990; Iinuma et al, 1995; Blant et al, 1996). In the present study not only was the 30 mW $cm^{-2}$ fluence rate superior to the higher fluence rates at equal total fluence (100 J $cm^{-2}$), but even lower fluences produced more lasting tumour responses, implying markedly enhanced PDT efficiency.

At least two mechanisms may be responsible for the fluence rate-dependent differences in anti-tumour activity. First, the increased oxygen availability throughout the tumour tissue during illumination at low fluence rate seems to lead to a modest, but statistically significant increase in direct photodynamic tumour cell kill in this tumour model (Sitnik and Henderson, 1997). Similarly, increased fluence rate-dependent cytotoxicity was reported by Foster et al (1993) for multi-cell tumour spheroids in vitro. Second, the severe disruption of the microvascular perfusion of the tumour-surrounding skin at low-fluence-rate treatment may delay the re-supply of oxygen and nutrition to any PDT-surviving tumour cells and so retard tumour regrowth.

The observations reported here may be of significant clinical importance if it can be shown that lower fluence rate does not adversely affect therapeutic ratio. Preclinical studies examining the influence of fluence rate on PDT exposure of normal tissue, especially the skin, are currently underway, as is an extensive clinical study determining tumour $pO_2$ during PDT in patients undergoing treatment of neoplastic skin lesions.

### ABBREVIATIONS

HBSS, Hanks' balanced salt solution; PDT, photodynamic therapy; PDT MODeM, PDT molecular oxygen-depletion model; RIF, radiation-induced fibrosarcoma; RPCI, Roswell Park Cancer Institute.

### ACKNOWLEDGEMENTS

We thank Dr William Greco, Department of Biomathematics, RPCI, for providing statistical advice. This work was supported by National Cancer Institute (NCI) grants CA42278, CA55791 and CA16056.

### REFERENCES

Bellnier DA, Potter WR, Vaughan LA, Parsons JC, Greco WR and Henderson BW (1995) The validation of a new vascular damage assay for photodynamic therapy agents. *Photochem Photobiol* **62**: 896–905

Bellnier DA, Greco WR, Parsons JC, Oseroff AR, Kuebler A and Dougherty TJ (1997) An assay for the quantitation of Photofrin[R] in tissues and fluids. *Photochem Photobiol* **66**(2): 237–244

Blant SA, Woodtli A, Wagnieres G, Fontolliet C, Van den Bergh H and Monnier P (1996) *In vivo* fluence rate effect in photodynamic therapy of early cancers with tetra(m-hydroxyphenyl) chlorin. *Photochem Photobiol* **64**: 963–968

BMDP (1990) *Statistical Software Manual*. pp. 739–768

Chen Q, Chen H and Hetzel FW (1996) Tumor oxygenation changes post-photodynamic therapy. *Photochem Photobiol* **63**: 128–131

Fenton BM and Way BA (1993) Vascular morphometry of KHT and RIF-1 murine sarcomas. *Radiother Oncol* **28**: 57–62

Fingar VH, Wieman TJ, Park YJ and Henderson BW (1992*a*) Implications of a pre-existing tumor hypoxic fraction on photodynamic therapy. *J Surg Res* **53**: 524–528

Fingar VH, Wieman TJ, Wiehle SA and Cerrito PB (1992*b*) The role of microvascular damage in photodynamic therapy: the effect of treatment on vessel constriction, permeability, and leukocyte adhesion. *Cancer Res* **52**: 4914–4921

Foster TH and Nichols MG (1995) Oxygen sensitivity of PDT determined from time-dependent electrode measurements in spheroids. In *Optical Methods for Tumor Treatment and Detection: Mechanisms and Techniques in Photodynamic Therapy IV*, Dougherty TJ. (ed.), pp. 141–151. SPIE Press: Bellingham, WA

Foster TH, Murant RS, Bryant RG, Knox RS, Gibson SL and Hilf R (1991) Oxygen consumption and diffusion effects in photodynamic therapy. *Radiat Res* **126**: 296–303

Foster TH, Hartley DF, Nichols MG and Hilf R (1993) Fluence rate effects in photodynamic therapy of multicell tumor spheroids. *Cancer Res* **53**: 1249–1254

Georgakoudi I, Nichols MG and Foster TH (1997) The mechanism of Photofrin photobleaching and its consequences for photodynamic dosimetry. *Photochem Photobiol* **65**: 135–144

Gibson SL, VanDerMeid KR, Murant RS, Raubertas RF and Hilf R (1990) Effects of various photoradiation regimens on the antitumor efficacy of photodynamic therapy for R3230AC mammary carcinomas. *Cancer Res* **50**: 7236–7241

Gomer CJ and Razum NJ (1984) Acute skin response in albino mice following porphyrin photosensitization under oxic and anoxic conditions. *Photochem Photobiol* **40**: 435–439

Henderson BW and Dougherty TJ (1992) How does photodynamic therapy work? *Photochem Photobiol* **55**: 145–157

Henderson BW and Fingar VH (1987) Relationship of tumor hypoxia and response to photodynamic treatment in an experimental mouse tumor. *Cancer Res* **47**: 3110–3114

Henderson BW and Fingar VH (1989) Oxygen limitation of direct tumor cell kill during photodynamic treatment of a murine tumor model. *Photochem Photobiol* **49**: 299–304

Henning JP, Fournier RL and Hampton JA (1995) A transient mathematical model of oxygen depletion during photodynamic therapy. *Radiat Res* **142**: 221–226

Honess D, Laurence V, Ward R, Shaw J and Bleehen N (1995) The effects of nicotinamide and carbogen, individually or in combination, on RIF-1 tumour oxygenation. In *Tumor Oxygenation*, Vaupel PW, Kelleher DK and Gunderoth M. (eds), pp. 137–144. Gustav Fischer: Stuttgart

Horsman MR, Khalil AA, Siemann DW, Grau C, Hill SA, Lynch EM, Chaplin DJ and Overgaard J (1994) Relationship between radiobiological hypoxia in tumors and electrode measurements of tumor oxygen. *Int J Radiat Oncol Biol Phys* **29**: 439–442

Iinuma S, Wagnieres G, Schomacker KT, Bamberg M and Hasan T (1995) The importance of fluence rate in photodynamic therapy with ALA-induced PpIX and BPD-MA in a rat bladder tumor model. In *Optical Methods for Tumor Treatment and Detection: Mechanisms and Techniques in Photodynamic Therapy IV*, Dougherty TJ. (ed.), pp. 136–140. SPIE Press: Bellingham, WA

Kavanaugh M, Sun A, Hu Q and Hill RP (1996) Comparing techniques of measuring tumor hypoxia in different murine tumors: Eppendorf $pO_2$ histograph, [$^3$H]misonidazole binding and paired survival assay. *Radiat Res* **145**: 491–500

Lyng H, Skretting A and Rofstad EK (1992) Blood flow in six human melanoma xenograft lines with different growth characteristics. *Cancer Res* **52**: 584–592

Mantel N (1966) Evaluation of survival data and two new rank order statistics arising in its consideration. *Cancer Chemother Rep* **50**: 163–170

Moan J and Sommer S (1985) Oxygen dependence of the photosensitizing effect of hematoporphyrin derivative in NHIK 3025 cells. *Cancer Res* **45**: 1608–1610

Nichols MG and Foster TH (1994) Oxygen diffusion and reaction kinetics in the photodynamic therapy of multicell tumour spheroids. *Phys Med Biol* **39**: 2161–2181

Roberts DJH, Cairnduff F, Driver I, Dixon B and Brown SB (1994) Tumour vascular shutdown following photodynamic therapy based on polyhaematoporphyrin or 5-aminolaevulinic acid. *Int J Oncol* **5**: 763–768

Rofstad EK, Fenton BM and Sutherland RM (1988) Intracapillary $HbO_2$ saturations in murine tumours and human tumour xenografts measured by cryospectrophotometry: relationship to tumour volume, tumour pH and fraction of radiobiologically hypoxic cells. *Br J Cancer* **57**: 494–502

Sitnik TM and Henderson BW (1997) Effects of fluence rate on cytotoxicity during photodynamic therapy. In *Optical Methods for Tumor Treatment and Detection: Mechanisms and Techniques in Photodynamic Therapy IV*, Dougherty TJ. (ed.), pp. 95–102. SPIE Press: Bellingham, WA

Stone HB, Brown JM, Phillips TL and Sutherland RM (1993) Oxygen in human tumors: correlations between methods of measurement and response to therapy. *Radiat Res* **136**: 422–434

Tromberg BJ, Orenstein A, Kimel S, Barker SJ, Hyatt J, Nelson JS and Berns MW (1990) In vivo tumor oxygen tension measurements for the evaluation of the efficiency of photodynamic therapy. *Photochem Photobiol* **52**: 375–385

Twentyman PR, Brown JM, Gray JW, Franko AJ, Scoles MA and Kallman RF (1980) A new mouse tumor model system (RIF-1) for comparison of end-point studies. *J Natl Cancer Inst* **64**: 595–604

© Cancer Research Campaign 1998

van Geel IPJ, Oppelaar H, Oussoren YG and Stewart FA (1994) Changes in perfusion of mouse tumours after photodynamic therapy. *Int J Cancer* **56**: 224–228

van Geel IPJ, Oppelaar H, Rijken PFJW, Bernsen HJJA, Hagemeier NEM, van der Kogel AJ, Hodgkiss RJ and Stewart FA (1996) Vascular perfusion and hypoxic areas in RIF-1 tumours after photodynamic therapy. *Br J Cancer* **73**: 288–293

Vaupel P, Kallinowski F and Okunieff P (1989) Blood flow, oxygen and nutrient supply, and metabolic microenvironment of human tumors: a review. *Cancer Res* **49**: 6449–6465

Vaupel P, Schlenger K, Knoop C and Höckel M (1991) Oxygenation of human tumors: evaluation of tissue oxygen distribution in breast cancers by computerized $O_2$ tension measurements. *Cancer Res* **51**: 3316–3322

*© Cancer Research Campaign 1998*